# Determinants of venous return in steady-state physiology and asphyxia-induced circulatory shock and arrest: an experimental study

**DOI:** 10.1186/s40635-022-00440-z

**Published:** 2022-04-12

**Authors:** Athanasios Chalkias, Eleni Laou, Nikolaos Papagiannakis, Giolanda Varvarousi, Dimitrios Ragias, Anastasios Koutsovasilis, Demosthenes Makris, Dimitrios Varvarousis, Nicoletta Iacovidou, Ioannis Pantazopoulos, Theodoros Xanthos

**Affiliations:** 1 Department of Anesthesiology, Faculty of Medicine, University of Thessaly, Larisa, Greece; 2 Outcomes Research Consortium, Cleveland, OH 44195 USA; 3 Hellenic Society of Cardiopulmonary Resuscitation, Athens, Greece; 4 1st Department of Neurology, Eginition University Hospital, Medical School, National and Kapodistrian University of Athens, Athens, Greece; 5 Division of Obstetrics, Department of Anesthesiology, Alexandra Hospital, Athens, Greece; 6 3rd Department of Internal Medicine, Nikaia General Hospital, Piraeus, Greece; 7 Department of Critical Care Medicine, Faculty of Medicine, University of Thessaly, Larisa, Greece; 8 Department of Cardiology, Asklepeion General Hospital, Athens, Greece; 9 Neonatal Department, Aretaieio Hospital, Medical School, National and Kapodistrian University of Athens, Athens, Greece; 10 Department of Emergency Medicine, Faculty of Medicine, University of Thessaly, Larisa, Greece; 11 School of Medicine, European University Cyprus, Nicosia, Cyprus; 12 Department of Anesthesiology, University Hospital of Larisa, Biopolis, 41110 Larisa, Greece

**Keywords:** Mean circulatory filling pressure, Venous return, Vascular capacitance hemodynamics, Shock, Resuscitation, Cardiac arrest, Hypoxemia, Hypercapnia

## Abstract

**Background:**

Mean circulatory filling pressure (Pmcf) provides information on stressed volume and is crucial for maintaining venous return. This study investigated the Pmcf and other determinants of venous return in dysrhythmic and asphyxial circulatory shock and arrest.

**Methods:**

Twenty Landrace/Large-White piglets were allocated into two groups of 10 animals each. In the dysrhythmic group, ventricular fibrillation was induced with a 9 V cadmium battery, while in the asphyxia group, cardiac arrest was induced by stopping and disconnecting the ventilator and clamping the tracheal tube at the end of exhalation. Mean circulatory filling pressure was calculated using the equilibrium mean right atrial pressure at 5–7.5 s after the onset of cardiac arrest and then every 10 s until 1 min post-arrest. Successful resuscitation was defined as return of spontaneous circulation (ROSC) with a MAP of at least 60 mmHg for a minimum of 5 min.

**Results:**

After the onset of asphyxia, a ΔPmca increase of 0.004 mmHg, 0.01 mmHg, and 1.26 mmHg was observed for each mmHg decrease in PaO_2_, each mmHg increase in PaCO_2,_ and each unit decrease in pH, respectively. Mean Pmcf value in the ventricular fibrillation and asphyxia group was 14.81 ± 0.5 mmHg and 16.04 ± 0.6 mmHg (*p* < 0.001) and decreased by 0.031 mmHg and 0.013 mmHg (*p* < 0.001), respectively, for every additional second passing after the onset of cardiac arrest. With the exception of the 5–7.5 s time interval, post-cardiac arrest right atrial pressure was significantly higher in the asphyxia group. Mean circulatory filling pressure at 5 to 7.5 s after cardiac arrest predicted ROSC in both groups, with a cut-off value of 16 mmHg (AUC = 0.905, *p* < 0.001).

**Conclusion:**

Mean circulatory filling pressure was higher in hypoxic hypercapnic conditions and decreased at a lower rate after cardiac arrest compared to normoxemic and normocapnic state. A Pmcf cut-off point of 16 mmHg at 5–7.5 s after cardiac arrest can highly predict ROSC.

**Supplementary Information:**

The online version contains supplementary material available at 10.1186/s40635-022-00440-z.

## Background

Mean circulatory filling pressure (Pmcf) is the equilibrium pressure throughout the circulation (systemic and pulmonary system) during circulatory arrest [1]. It provides information on intravascular effective blood volume, or stressed volume, and is inversely proportional to the compliance of the cardiovascular system [2]. The stressed volume is crucial for maintaining venous return, which forms the basis for heart–lung interactions in patients with altered vascular states and is the major determinant of cardiac output (CO) [3].

In patients with shock, vasoconstriction of the veins does not increase the impedance to the flow greatly, but shifts the blood volume downstream towards the heart. Mean circulatory filling pressure is determined by the volume and the elastic properties of the vasculature and thus may vary among different pathophysiological states. Therefore, monitoring of venous tone and knowing Pmcf would help to better understand the hemodynamic status at bedside. However, the lack of knowledge on the associated physiological mechanisms and the little evidence in critically ill patients have resulted to the underuse of Pmcf [4, 5].

Our research group has shown that significant differences exist between dysrhythmic and asphyxial cardiac arrest regarding their pathophysiologic pathways, metabolic disturbance, organ dysfunction, and response to therapy [6, 7]. We hypothesized that these alterations cause different effects on the driving force for venous return. In the present study, we aimed to elucidate this topic in greater depth. To this end, we investigated the Pmcf and other determinants of venous return following dysrhythmic and asphyxial circulatory shock-arrest in an experimental swine model.

## Methods

### Ethics approval

All animal procedures performed were approved by the Directorate of Veterinary Services according to the national legislation regarding ethical and experimental procedures. These procedures conformed to the guidelines from Directive 2010/63/EU of the European Parliament on the protection of animals used for scientific purposes or the current National Institutes of Health guidelines. This manuscript adheres to the applicable ARRIVE 2.0 guidelines [8].

### Study objectives

The primary objective was to investigate the difference in Pmcf change over time between dysrhythmic and asphyxial circulatory shock-arrest. Secondarily, we aimed to assess the correlation between Pmcf and return of spontaneous circulation (ROSC) in these states.

### Origin and source of the animals

This analysis included 20 healthy Landrace/Large-White piglets aged 10–12 weeks with average weight 20 ± 1 kg, all purchased from the same breeder (Validakis, Koropi, Greece). Taking into consideration the principles of 3R, which stand for Replacement, Reduction, and Refinement and represent a responsible approach for performing more humane animal research (https://www.nc3rs.org.uk/the-3rs), we included animals that were used for educative purposes in the Experimental Part of the Postgraduate Study Program “Resuscitation” of the University of Athens, Greece (permit no. 1188 and 4856).

One week prior to the experiments, the animals were transported to the research facility (Experimental-Research Center Elpen, European Ref Number EL 09 BIO 03) and were acclimatized to laboratory conditions, as previously described [9]. The day before the experimentation, the animals were fasted but had free access to water. All animals received anesthetic and surgical procedures in compliance with the Guide for the Care and Use of Laboratory Animals.

### Animal preparation

In brief, the animals were premedicated with intramuscular ketamine hydrochloride (Merial, Lyon, France) 10 mg/kg, midazolam (Roche, Athens, Greece) 0.5 mg/kg, and atropine sulphate (Demo, Athens, Greece) 0.05 mg/kg. The animals were subsequently transported to the operation research facility and intravascular access was obtained through the auricular veins. Induction of anesthesia was achieved with an intravenous bolus dose of propofol (Diprivan 1% w/v; AstraZeneca, Luton, United Kingdom) (2 mg/kg) and fentanyl (Janssen Pharmaceuticals, Beerse, Belgium) (2 μg/kg). The same researcher performed the intubation while the animals were breathing spontaneously with a size 6.0-mm cuffed endotracheal tube. The endotracheal tube was secured on the lower jaw, and successful intubation was ascertained by auscultation of both lungs while ventilated with a self-inflating bag.

The animals were then immobilized in the supine position on the operating table and were volume-controlled ventilated (tidal volume 15 ml/kg, *I*:*E* 1:2, PEEP 0 cm H_2_O, and FiO_2_ 0.21—Siare Alpha-Delta Lung Ventilator; Siare s.r.l. Hospital Supplies, Bologna, Italy) [10]. Additional 1 mg/kg propofol, 0.15 mg/kg cis-atracurium, and 4 μg/kg fentanyl were administered intravenously to ascertain synchrony with the ventilator. Propofol 0.1 mg/kg/min, cis-atracurium 20 μg/kg/min, and fentanyl 0.6 μg/kg/min were administered to maintain adequate anesthetic depth throughout the study [9, 11]. We used the assessment of jaw tone throughout the experiment to assess the anesthetic depth according to the guidelines on anesthesia and analgesia in swine [10]. Normocapnia was achieved using continuous monitoring of end-tidal CO_2_ (Tonocap TC-200-22-01; Engstrom Division, Instrumentarium Corp, Helsinki, Finland) and the respiratory rate was adjusted to maintain end-tidal carbon dioxide 35–40 mmHg. Pulse oximetry was monitored throughout the experiment. Body temperature was monitored by a rectal temperature probe and was maintained between 38.5 and 39.5 °C with a heating blanket [10].

Electrocardiographic monitoring was used using leads I, II, III, aVR, aVL, and aVF, which were connected to a monitor (Mennen Medical, Envoy; Papapostolou, Athens, Greece). The monitor electronically calculated the heart rate. For measurement of the aortic pressures, an arterial catheter (model 6523, USCI CR, Bart; Papapostolou) was inserted and forwarded into the descending aorta after surgical preparation of the right internal carotid artery. The systolic (SAP) and diastolic (DAP) arterial pressures were recorded, whereas mean arterial pressure (MAP) was determined by the electronic integration of the aortic blood pressure waveform. Then, the left internal jugular vein was cannulated and a Swan–Ganz catheter (Opticath 5.5F, 75 cm; Abbott, Ladakis, Athens, Greece) was inserted into the right atrium. Intravascular catheters were attached to pressure transducers that were aligned to the level of the right atrium and were calibrated before their use. This allowed the recording of right atrial pressure (*P*_RA_) and arterial pressures. In the dysrhythmic group, the right internal jugular vein was also surgically prepared, and a 5F flow-directed pacing catheter (Pacel, 100 cm; St Jude Medical, Ladakis, Athens, Greece) was advanced into the apex of the right ventricle.

A FloTrac sensor kit was connected to the arterial line and coupled to a Vigileo monitor (FloTrac/Vigileo; Edwards Lifescience, Irvine, California, USA) that allowed CO measurement. Systemic vascular resistance (SVR) was calculated using the formula SVR = (MAP − *P*_RA_)/CO × 80, as previously described [12, 13]. Coronary perfusion pressure (CPP) was electronically calculated as the difference between minimal DAP and the simultaneously measured right atrial diastolic pressure. Arterial blood gases (ABGs) were measured on a blood-gas analyzer (IRMA SL Blood Analysis System, Part 436301; Diametrics Medical Inc, Roseville, MN 55113). Baseline data were collected after allowing each animal to stabilize for 30 min.

### Calculation of baseline mean circulatory filling pressure analogue and related variables

Mean circulatory filling pressure analog (Pmca) was calculated after baseline measurements in order to assess the effective circulating volume and the driving pressure for venous return in stable cardiovascular conditions. The methods of the Pmca algorithm have been described in detail before [14–18]. Briefly, based on a Guytonian model of the systemic circulation [CO = VR = (Pmcf − *P*_RA_)/*R*_VR_], an analogue of Pmcf can be derived using the mathematical model Pmca = (*a* × *P*_RA_) + (*b* × MAP) + (*c* × CO), where *P*_RA_ is right atrial pressure and *R*_VR_ is resistance to venous return [19, 20]. In this formula, *a* and *b* are dimensionless constants (*a* + *b* = 1). Assuming a veno-arterial compliance ratio of 24:1, *a* = 0.96 and *b* = 0.04, reflecting the contribution of venous and arterial compartments, and c resembles arteriovenous resistance and is based on a formula including age, height and weight [20]:

$$c = \frac{{0.038 \left( {94.17 + 0.193 \times {\text{ age}}} \right)}}{{4.5 \left( {0.99^{{{\text{age}} - 15}} } \right) 0.007184 \cdot \left( {{\text{height}}^{0.725} } \right) \left( {{\text{weight}}^{0.425} } \right)}}$$

In addition, the following values were determined: (1) pressure gradient for venous return (PG_VR_) was defined as the pressure difference between Pmcf (or Pmca) and *P*_RA_ [PG_VR_ = Pmcf (or Pmca) − *P*_RA_]; (2) resistance to venous return was defined as the ratio of the pressure difference between Pmcf (or Pmca) and *P*_RA_ and cardiac output [*R*_VR_ = (Pmcf (or Pmca) − *P*_RA_)/CO]. This formula is used to describe venous return during transient states of imbalances [Pmcf (or Pmca) is the average pressure in the systemic circulation and R_VR_ is the resistance encountered to the heart] [21, 22]; and (3) Efficiency of the heart (Eh) was defined as the ratio of the pressure difference between Pmcf (or Pmca) and *P*_RA_ and Pmcf (or Pmca) [Eh = (Pmcf (or Pmca) − *P*_RA_)/Pmcf (or Pmca)]. This equation was proposed for the measurement of heart performance, and during the cardiac stop ejection, *P*_RA_ is equal to the Pmcf (or Pmca) and Eh approaches zero [17, 23].

### Experimental protocols

After baseline data were collected, the swine were allocated into two groups of 10 animals each, the dysrhythmic and asphyxia group.

#### Dysrhythmic group

Ventricular fibrillation was induced with a 9 V ordinary cadmium battery via a pacing wire forwarded into the right ventricle through the exposed right jugular vein, as previously described [9]. All animals suffered ventricular fibrillation with the first attempt.

#### Asphyxia group

Asphyxia was induced by stopping and disconnecting the ventilator and clamping the tracheal tube at the end of exhalation [7]. During the asphyxia interval, we observed an initial increase in heart rate (peaking at 2 min) and MAP (peaking at 3 min) which progressively declined until cardiac arrest occurred after 8.1 ± 1.2 min from endotracheal tube clamping. Full muscle paralysis prevented any form of gasping that would be anticipated because of hypoxic injury.

### Cardiac arrest interval

In both groups, cardiac arrest was recognized electrocardiographically and/or confirmed by a sudden drop in MAP and loss of arterial pulse. Mechanical ventilation in the ventricular fibrillation group and administration of anesthetics in both groups were discontinued simultaneously with the onset of cardiac arrest. All animals were left untreated for 4 min.

At the onset of asphyxial cardiac arrest, 2 animals had ventricular fibrillation, 1 had asystole, and 7 had pulseless electrical activity. However, significant rhythm changes were noted during the 4-min period of untreated arrest. At the end of the fourth minute, 6 animals had ventricular fibrillation, 1 animal had asystole, and 3 animals had pulseless electrical activity. At this time, all animals in this group had profound hypoxemia with hypercapnia and respiratory acidosis.

### Calculation of mean circulatory filling pressure

Shortly after cardiac arrest, significant changes in vasomotor tone occur, while the arterial pressure falls and the venous pressure rises until they almost reach equilibrium [24, 25]. These mandate the measurement of Pmcf to be made within the first few seconds after arrest [24, 26]. However, the hypotension-induced baroreflex withdrawal maintains an antegrade and pulmonary blood flow that may continue for more than 30–60 s [25]. As Pmcf may vary among individuals, the maximum flow could be better assessed if the time of arrest is more than 20 s [27, 28].

Based on the aforementioned, we initially measured Pmcf using the equilibrium mean *P*_RA_ between 5 and 7.5 s after the onset of cardiac arrest, before the reflex response significantly alters the measured plateau pressure [25, 29, 30]. Then, we continued measuring Pmcf every 10 s until 1 min post-cardiac arrest, provided that the measured plateau pressure had not significantly altered. In this study, Pmcf was measured at six time points (5–7.5 s, 15–17.5 s, 25–27.5 s, 35–37.5 s, 45–47.5 s, and 55–57.5 s).

As arteries are much less compliant than veins, transfer of the remaining arterial volume sufficient to equalize pressures throughout the vasculature could not significantly increase Pmcf or affect measurements in our study [29]. In this context, a plateau was considered adequate to allow accurate measurement if mean *P*_RA_ rose by less than 1 mmHg over the period from 5 to 7.5 s after the onset of cardiac arrest [29]. In the present study, all animals had adequate plateau and were included for further analysis.

### Cardiopulmonary resuscitation

**Fig. 1:**
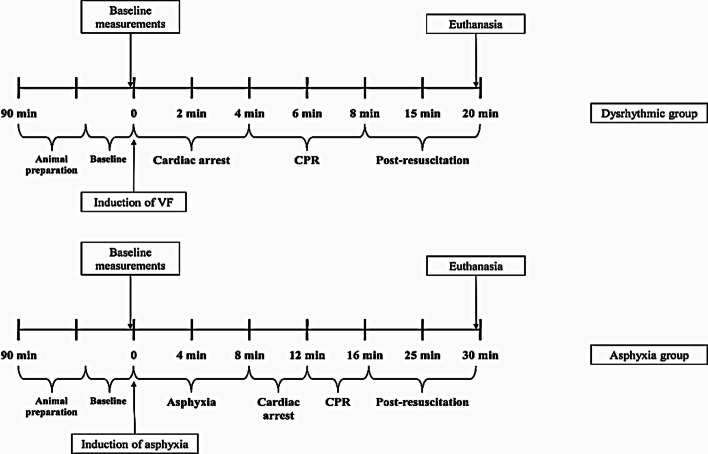
Experimental protocol outline. *VF* ventricular fibrillation, *CPR* cardiopulmonary resuscitation

The animals were resuscitated according to the 2015 European Resuscitation Council Guidelines for Resuscitation with ventilation in 100% oxygen and chest compressions at a rate of 100/min (LUCAS 2 CPR device, Jolife, Lund, Sweden) [31]. No drugs were administered during resuscitation. All animals were resuscitated by the same researchers, while successful resuscitation was defined as ROSC with a MAP of at least 60 mmHg for a minimum of 5 min (Fig. 1).

### Statistical analysis

Statistical analysis was performed using R v4.1. Spearman’s method was used to correlate hemodynamic measurements with Pmca at baseline. The Benjamini–Hochberg false discovery rate correction was applied in the resulting *p*-values to account for the multiple number of comparisons. Adjusted *p*-values less than 0.05 were deemed significant. Linear mixed effects (LME) models were used in order to assess the effect of time after cardiac arrest in Pmcf. Time after cardiac arrest and intervention group were included as fixed factors and the different subjects (piglets) was included as random factor. Receiver operating characteristic (ROC) curve analysis was used to assess the prognostic value of Pmcf and the respective model for ROSC. DeLong’s method will be used for the computation of asymptotic *p*-values for difference of area under the curve (AUC) from the theoretical area of 0.5. Optimal cut-off values were estimated by maximizing Youden’s index.

## Results

### Baseline measurements

**Fig. 2:**
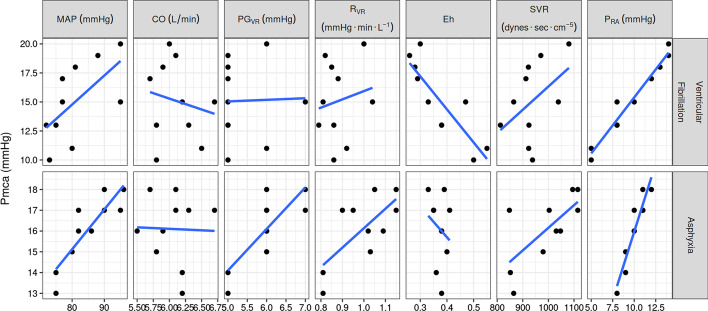
Scatter plots of Pmca at baseline with MAP, CO, PG_VR_, *R*_VR_, Eh, SVR, and *P*_RA_ in the ventricular fibrillation and asphyxia groups. MAP: mean arterial pressure; CO: cardiac output; PG_VR_: pressure gradient for venous return; *R*_VR_: resistance to venous return; Eh: efficiency of the heart; SVR: systemic vascular resistance; *P*_RA_: right atrial pressure

Baseline parameters in both groups are depicted in Tables 1 and 2 and in Fig. 2. Baseline Pmca was strongly correlated with *P*_RA_ both in the ventricular fibrillation (*ρ* = 0.975, *p* < 0.001) and asphyxia group (*ρ* = 0.940, *p* < 0.001).

**Table 1:** Systemic hemodynamic parameters and determinants of venous return at the end of the baseline period

	VF (mean)	Asphyxia (mean)	Difference (95% confidence interval)	Adjusted *p*-value
Heart rate (bmp)	83.1	86.8	3.7 (− 1.39–8.79)	0.461
Systolic arterial pressure (mmHg)	102.6	115.1	12.5 (5.11–19.89)	0.036*
Diastolic arterial pressure (mmHg)	67.8	69.9	2.1 (− 6.8–11)	0.847
Mean arterial pressure (mmHg)	81.3	85.1	3.8 (− 3.77–11.37)	0.611
Systemic vascular resistance (dynes⋅s⋅cm^−5^)	938.6	994.8	56.2 (− 31.23–143.71)	0.494
Right atrial pressure (mmHg)	9.7	10.1	0.4 (− 2.02–2.82)	0.847
Cardiac output (L⋅min^−1^)	6.1	6.05	− 0.05 (− 0.36–0.26)	0.847
Coronary perfusion pressure (mmHg)	58.1	59.8	1.7 (− 6.2–9.6)	0.847
Pulse pressure (mmHg)	34.8	45.2	10.4 (− 0.76–21.56)	0.264
Shock index	0.82	0.77	− 0.05 (− 0.13–0.03)	0.494
Modified shock index	1.04	1.03	− 0.01 (− 0.11–0.09)	0.892
Mean circulatory filling pressure analogue (mmHg)	15.1	16.1	1 (− 1.5–3.5)	0.733
Pressure gradient for venous return (mmHg)	5.4	6	0.6 (− 0.04–1.24)	0.264
Resistance to venous return (mmHg min L^−1^)	0.883	0.996	0.113 (0.01–0.21)	0.224
Efficiency of the heart	0.374	0.373	− 0.001 (− 0.07–0.07)	0.976
End-tidal carbon dioxide (mmHg)	37.4	37.9	0.5 (− 2.28–3.28)	0.847

*VF* ventricular fibrillation

*p < 0.05

**Table 2:** Correlation of mean circulatory filling pressure analogue with systemic hemodynamics at the end of baseline period

Group	rho	Adjusted *p*-value*
Ventricular fibrillation		
Mean arterial pressure	0.699	0.068
Cardiac output	− 0.107	0.852
Pressure gradient for venous return	0.068	0.852
Resistance to venous return	0.07	0.852
Efficiency of the heart	− 0.862	0.006
Systemic vascular resistance	0.299	0.625
Right atrial pressure	0.975	< 0.001
Asphyxia		
Mean arterial pressure	0.891	0.004
Cardiac output	− 0.071	0.852
Pressure gradient for venous return	0.757	0.039
Resistance to venous return	0.63	0.116
Efficiency of the heart	− 0.213	0.776
Systemic vascular resistance	0.616	0.116
Right atrial pressure	0.940	< 0.001

^*^Adjusted *p*-values with Benjamini–Hochberg correction

### Cardiac arrest interval

#### Mean circulatory filling pressure changes

Mean Pmcf value in the ventricular fibrillation and asphyxia group was 14.81 ± 0.5 mmHg and 16.04 ± 0.6 mmHg, respectively (*p* < 0.001). No statistically significant correlation was observed between Pmca and Pmcf in both groups (ventricular fibrillation: rho − 0.229, *p* = 0.52; asphyxia: rho 0.310, *p* = 0.28).

**Fig. 3:**
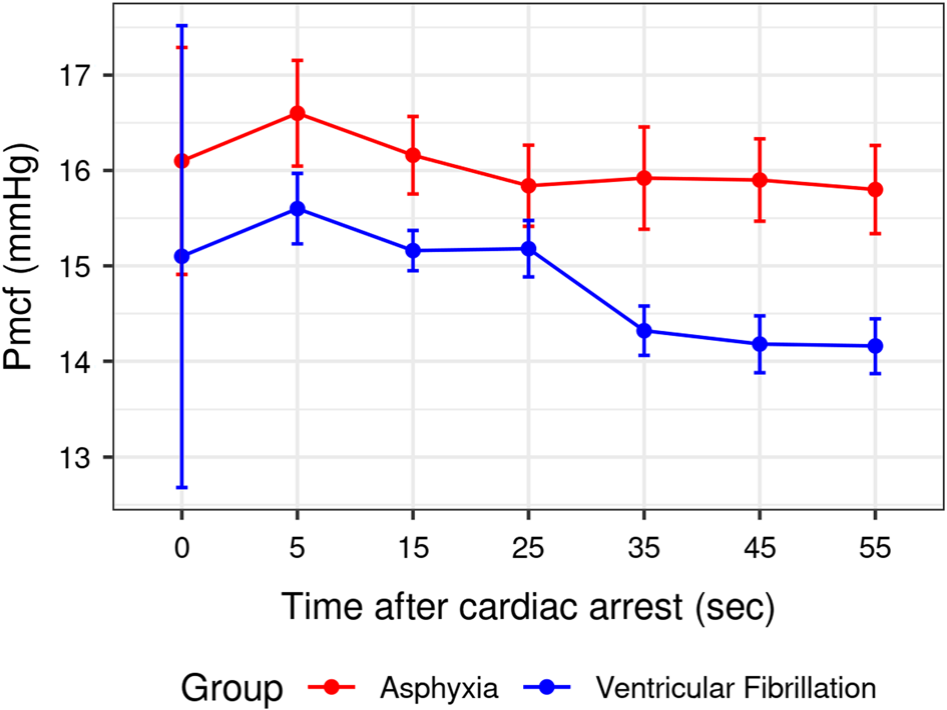
Post-cardiac arrest Pmcf fluctuation in the ventricular fibrillation and asphyxia group. The difference was statistically significant at all time points (*p* < 0.001)

In both groups, an initial increase in Pmcf was observed immediately after the onset of cardiac arrest followed by a significant decrease with time (*p* < 0.001). Post-cardiac arrest Pmcf was constantly lower in the ventricular fibrillation group compared to the asphyxia group (*p* < 0.001, Fig. 3). Specifically, Pmcf decreased by 0.031 mmHg and 0.013 mmHg for every additional second passing after the onset of cardiac arrest in the ventricular fibrillation and asphyxia group, respectively (*p* < 0.001).

#### Changes in other parameters of venous return

**Fig. 4:**
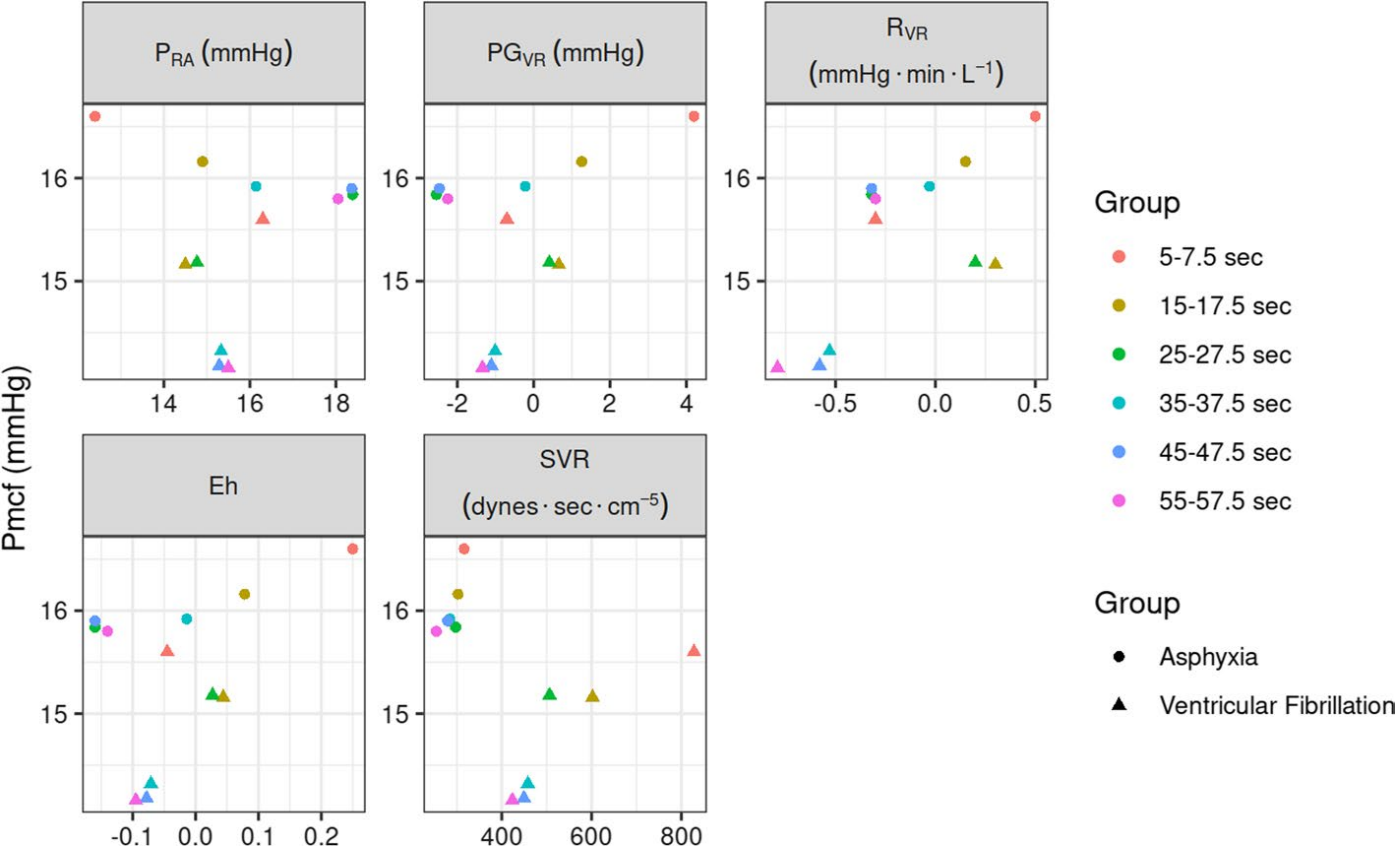
Scatter plots of average Pmcf in the *y*-axis after cardiac arrest. In the *x*-axis the mean *P*_RA_, PG_VR_, *R*_VR_, Eh, and SVR for each time-point after cardiac arrest was plotted. Dots represent observations in the asphyxia group, while triangles represent observations in the ventricular fibrillation group. The different colors show the respective time-point the average value represents. No statistically significant linear regression model was evident between Pmcf (as dependent variable) and the other variables as independent. *P*_RA_, right atrial pressure; PG_VR_, pressure gradient for venous return; *R*_VR_, resistance to venous return; Eh, efficiency of the heart; SVR, systemic vascular resistance

Right atrial pressure increased after cardiac arrest in the asphyxia group, but decreased in the ventricular fibrillation group. With the exception of the 5- to 7.5-s time interval, *P*_RA_ was significantly higher in the asphyxia group after cardiac arrest. The respective *p*-values for each time-point are 5–7.5 s, *p* < 0.001; 15–17.5 s, *p* = 0.535; 25–27.5 s, *p* < 0.001; 35–37.5 s, *p* = 0.127; 45–47.5 s, *p* < 0.001; and 55–57.5 s, *p* < 0.001 (Additional file 1: Fig. S1). Figure 4 presents the fluctuation of Pmcf over different time periods in association with PG_VR_, *R*_VR_, Eh, SVR, and *P*_RA_ in both groups.

#### Effects of progressing hypoxia and hypercapnia on mean circulatory filling pressure analogue gradient

Significant hemodynamic changes were observed with progressing hypoxia and hypercapnia. Specifically, Pmca was increased by 0.004 mmHg for each mmHg decrease in PaO_2_ and by 0.01 mmHg for each mmHg increase in PaCO_2_. Also, Pmca increased by 1.26 for each unit decrease in pH (Additional file 2: Fig. S2).

### Secondary outcomes

**Fig. 5:**
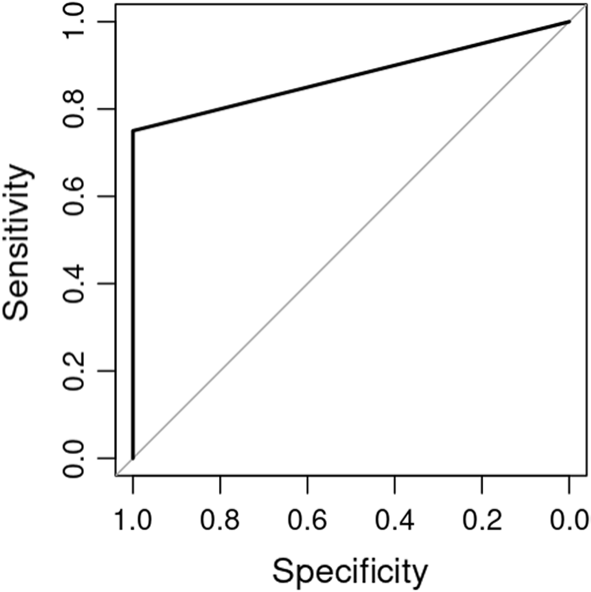
ROC curve for Pmcf at 5–7.5 s after cardiac arrest as a predictor of ROSC (AUC = 0.905, *p* < 0.001)

The differences in ABGs before the onset of resuscitation between the two groups are depicted in Table 3. Eight (80%) ventricular fibrillation animals and 8 (80%) asphyxia animals achieved ROSC. Mean circulatory filling pressure at 5 to 7.5 s after cardiac arrest predicted ROSC in both groups, with an area under the curve (AUC) equal to 0.905 (*p* < 0.001, Fig. 5). A Pmcf value ≥ 16 mmHg was indicative of ROSC, with a positive predictive value of 100% (95% CI 66.4% to 100%) and a negative predictive value of 50% (95% CI 23.2% to 100%), while lower Pmcf values were indicative of unsuccessful resuscitation. Also, Pmcf measured at 55 to 57.5 s after cardiac arrest had smaller discriminatory power for prediction of ROSC with an AUC equal to 0.718 (*p* = 0.153).

**Table 3:** Differences in respiratory and metabolic parameters before the onset and at the end of cardiac arrest interval

	Before the onset of cardiac arrest			At the end of cardiac arrest		
	VF group	Asphyxia group	*p*-value	VF group	Asphyxia group	*p*-value
pH	7.41 ± 0.04	7.42 ± 0.02	0.489	7.34 ± 0.03	7.1 ± 0.02	< 0.001
PaO_2_ (mmHg)	151 ± 6	152 ± 7	0.735	63 ± 3	17.9 ± 3	< 0.001
PaCO_2_ (mmHg)	42 ± 1.4	43 ± 1.6	0.154	46 ± 2	88 ± 7	< 0.001
Lactate (mmol L^−1^)	1.2 ± 0.2	1 ± 0.1	0.011	3 ± 0.3	16 ± 0.8	< 0.001

VF: ventricular fibrillation; PaO_2_: arterial partial pressure of oxygen; PaCO_2_: arterial partial pressure of carbon dioxide

## Discussion

Our study has revealed significant hemodynamic differences between the dysrhythmic and asphyxial circulatory shock state. After the onset of asphyxia, a ΔPmca increase of 0.004 mmHg, 0.01 mmHg, and 1.26 mmHg was observed for each mmHg decrease in PaO_2_, each mmHg increase in PaCO_2_, and each unit decrease in pH, respectively. Mean Pmcf in the ventricular fibrillation and asphyxia group was 14.81 ± 0.5 mmHg and 16.04 ± 0.6 mmHg, respectively. Post-cardiac arrest Pmcf decreased by 0.031 mmHg and 0.013 mmHg for every additional second in the ventricular fibrillation and asphyxia group, respectively, with the difference being significant at all time points. With the exception of the 5- to 7.5-s time interval, *P*_RA_ was significantly higher in the asphyxia group. A Pmcf cut-off point of 16 mmHg at 5–7.5 s after cardiac arrest highly predicted ROSC with an AUC of 0.905, but the discriminatory power decreased with time. Also, lower Pmcf values were indicative of unsuccessful resuscitation.

### Pathophysiological differences between dysrhythmic and asphyxial cardiac arrest

The hemodynamic and metabolic changes occurring after dysrhythmic and asphyxial cardiac arrest differ significantly. Dysrhythmic cardiac arrest leads to sudden and complete cessation of blood flow, with the fibrillating myocardium initially maintaining its energy stores and the cells not having serious damage. However, the no-flow state with global ischemia rapidly depletes high-energy phosphates within a few minutes [6]. On the other hand, asphyxial cardiac arrest is characterized by a prolonged time course and an important pre-arrest period where hypoxia and hypercapnia progressively advance along with maintained but gradually deteriorating cardiopulmonary function [6, 32]. During the asphyxial interval, general de-energization and mitochondrial complexes inactivation take place in parallel with oxygen shortage, eventually resulting in complete decompensation and a higher degree of energy depletion compared with dysrhythmic cardiac arrest [7, 33].

### Changes in mean circulatory filling pressure after asphyxial cardiac arrest

Although the loss of effective circulation in both groups caused an initial transient increase in Pmcf in parallel with the reflexly increase in pulmonary vascular resistance and *P*_RA_, Pmcf decreased at a slower rate and was significantly higher in the asphyxia group at all time points. In these animals, the progressive hypoxemic hypercapnia and acidosis caused a marked decrease of the vascular capacitance system, which significantly increased Pmcf via translocation of blood volume to the fast transit time peripheral systemic circuit (autotransfusion) [27, 34–36]. Specifically, a ΔPmca increase of 0.004 mmHg, 0.01 mmHg, and 1.26 mmHg was observed for each mmHg decrease in PaO_2_, each mmHg increase in PaCO_2_, and each unit decrease in pH, respectively (Fig. 3). Considering that these effects appear to be additive [34], the total body capacitance system response (reflexly or direct) to hypoxemic hypercapnic acidosis seems to induce a ΔPmca increase of 1.274 mmHg, which indeed is similar to the difference in mean Pmcf value between the two groups (1.23 mmHg). Other research groups have shown an increase of 0.036–0.068 mmHg of Pmcf per mmHg PaCO_2_ in dogs with hypoxic hypercapnia [34, 35, 37]. The difference between the species might be explained by the order of ranking the component regions of the splanchnic circulation with regard to function as a blood reservoir, which may be specific for the dog, and the sequestration of blood within the canine splanchnic circulation [2, 38, 39].

### Clinical implications—hemodynamic management of acute respiratory failure

The findings of the present study may be important for the hemodynamic management of acute respiratory failure in clinical practice. The acute alterations in pulmonary vascular resistance can strongly affect right ventricular afterload and *P*_RA_, especially during mechanical ventilation [40], and maintaining an effective circulation is crucial for tissue perfusion. Two questions that arise are: (a) whether the increase in Pmcf during hypoxemic hypercapnia is adequate for maintaining venous return in normovolemic patients with increased pulmonary vascular resistance; and if yes, (b) what are the possible side effects of exogenous vasopressor administration. In our study, the endotracheal tube was clamped at end-expiration at functional residual capacity (lowest pulmonary vascular resistance during the respiratory cycle), but the continuous emptying of the lungs (after the clamping) and the effects of anesthetics further reduced functional residual capacity, which is associated with pulmonary vascular resistance and *P*_RA_ rises [40, 41]. However, this may not be so important because cardiac preload is usually preserved despite substantial alterations in right ventricular afterload [42]. Also, Hoka et al. showed that coronary venous outflow increases by 400% and splanchnic venous outflow decreases by 60% after 3.5 min of hypoxic hypercapnia [39]. The activation of the abundant alpha-adrenergic receptors in the hepatic veins increases the impedance of the outflow of blood from the splanchnic system into systemic circulation and leads to sequestration of blood within the liver [2]. Based on our findings, the exogenous administration of an alpha-1 adrenergic agonist in patients with increased Pmcf not only seems meaningless, but will further enhance the constriction of arteries that supply blood to veins of relatively low compliance, leading to a decrease in flow through the whole circuit, venous return, and CO [2, 43]. Compared to alpha-1 adrenergic agonists, norepinephrine might exert an additional benefit by stimulation of beta-2 adrenoceptors, facilitating emptying of the venous system, but may also decrease pulmonary vascular compliance. The dynamic effects of hypoxic hypercapnia on the intra- and extrasplanchnic vascular capacitance system, together with the findings of the present study (increase in Pmcf—stressed volume), indicate that the main therapeutic interventions in these patients should focus on the alleviation of right ventricular afterload (pulmonary vasodilation, inotropic support) rather than on the administration of vasoconstrictors that can impair both venous return and right and left ventricular afterload [44, 45].

### Clinical implications—resuscitation of asphyxial cardiac arrest and the need for personalized physiology-guided treatment

The aforementioned may also apply in the resuscitation of asphyxial cardiac arrest, the majority of which result from respiratory failure secondary to airway obstruction, and with the initial rhythm usually being asystole or pulseless electrical activity [46]. Although guidelines on Resuscitation continue to suggest the administration of adrenaline as soon as possible in cardiac arrest with a non-shockable rhythm [47], evidence from some studies show that administration of exogenous epinephrine within the first 10–15 min after asphyxial cardiac arrest offers no hemodynamic or survival benefit [48, 49], while its adverse effects may persist for at least 30 min post-ROSC [25, 50]. In the present study, we found a higher Pmcf in the asphyxia group and that Pmcf at 5–7.5 s after cardiac arrest can highly predict ROSC, with a cut-off point of 16 mmHg in both groups. These indicate a preserved stressed volume and that the endogenous catecholamines seem sufficient for achieving ROSC [51]. Based on the findings of the present and other studies [48, 50–52], the inadequacy of global hemodynamic parameters to monitor tissue perfusion and oxygenation [53], and the significant possibility for spontaneous conversion of a non-shockable rhythm to a shockable one during the asphyxial cardiac arrest interval [33], the recommendation for administration of adrenaline as soon as possible in asphyxial cardiac arrest with a non-shockable rhythm should be critically re-evaluated, while Pmcf must be further studied as a new prognostic parameter for ROSC regardless of the cause of arrest.

The available evidence together with the findings of the present study support a physiology-guided treatment strategy to titrate the resuscitation efforts to patient’s physiological response. We have recently proposed such an approach, the “personalized physiology-guided resuscitation in highly monitored patients with cardiac arrest—PERSEUS resuscitation strategy”, which includes a protocolized administration of vasopressors to enhance volume recruitment from the unstressed compartment [54] and is currently investigated in a randomized controlled trial (ClinicalTrials.gov Identifier: NCT04428060). According to PERSEUS strategy, the effectiveness of chest compressions depends on venous return, which is proportional to the pressure gradient between Pmcf and central venous pressure (CVP), and particular attention is needed by the rescuers to determine peri-arrest Pmcf and necessarily prior to the onset of chest compressions. Ideally, the Pmcf must be measured immediately post-arrest, i.e., the equilibrium CVP at 5–7.5 s after cardiac arrest. Alternatively, the pre-arrest Pmca can be also used if for any reason the post-arrest Pmcf was not recorded. The optimization of Pmcf is crucial for maintaining DAP ≥ 40 mmHg during resuscitation and administration of vasopressors is based on these parameters [54]. Apart from the valuable information that Pmcf can provide about the function of the cardiovascular system, the sudden onset of cardiac arrest is another reason for considering the continuous monitoring of Pmca as of paramount importance.

## Limitations

We certainly acknowledge that the experiment was performed on apparently healthy animals and that the use of anesthetics may have impaired cardiovascular function and the response to stress [55]. Despite the closeness of the resemblance between the hemodynamics and biochemical/metabolic characteristics in response to injury of Landrace–Large-White swine and those of humans [56], the results of the present study may be different in older swine. In the present study, we included animals that were used for educative purposes in the Experimental Part of the Postgraduate Study Program “Resuscitation” of the University of Athens and thus, we had to use our established models. Per protocol, we do not insert a pacing catheter in the asphyxia group and thus, surgical trauma and possible effects of the catheter itself may not be the same in both groups. In addition, we were not able to digitally analyze the hemodynamic data into an electronic software. Nevertheless, our measurements are similar to those reported in literature. Furthermore, we measured Pmcf without addressing passive recoil effects or volume transfers between the peripheral and central compartments. However, our animals were normovolemic and our approach does not alter the central message that Pmcf is higher in hypoxic hypercapnia and decreases at a lower rate after cardiac arrest compared to normoxemic and normocapnic state. A future study would need to assess the possible impact of static airway pressure on Pmcf and to compare zero-flow measurements taken at varying levels of airway pressure.

## Conclusions

Mean circulatory filling pressure was higher in hypoxic hypercapnic conditions and decreased at a lower rate after cardiac arrest compared to normoxemic and normocapnic state. A Pmcf cut-off point of 16 mmHg at 5–7.5 s after cardiac arrest can highly predict ROSC.

## Supplementary Information

**Additional file 1: Figure S1.** Changes in post-cardiac arrest right atrial pressure with time in the asphyxia and ventricular fibrillation group. *P*_RA_, right atrial pressure.

**Additional file 2: Figure S2.** Comparison of baseline (Pmca) and post-cardiac arrest mean circulatory filling pressure in the asphyxia group demonstrating the significant effects of progressing hypoxic hypercapnia. Pmcf, mean circulatory filling pressure; PaO_2_, arterial partial pressure of oxygen; PaCO_2_, arterial partial pressure of carbon dioxide.

## Data Availability

Data can be made available upon request after publication through a collaborative process. Researchers should provide a methodically sound proposal with specific objectives in an approval proposal. Please contact the corresponding author for additional information.

## Abbreviations

Pmcf: Mean circulatory filling pressure
CO: Cardiac output
ROSC: Return of spontaneous circulation
SAP: Systolic arterial pressure
DAP: Diastolic arterial pressure
MAP: Mean arterial pressure
*P*_RA_: Right atrial pressure
SVR: Systemic vascular resistance
CPP: Coronary perfusion pressure
ABGs: Arterial blood gases
Pmca: Mean circulatory filling pressure analog
PGV_R_: Pressure gradient for venous return
*R*_VR_: Resistance to venous return
Eh: Efficiency of the heart
