# Supplementary figures and images for: Determinants of venous return in steady-state physiology and asphyxia-induced circulatory shock and arrest: an experimental study
Source: Intensive Care Med Exp. 2022 Apr 12;10:13. doi: 10.1186/s40635-022-00440-z (PMC9005574; doi:10.1186/s40635-022-00440-z)

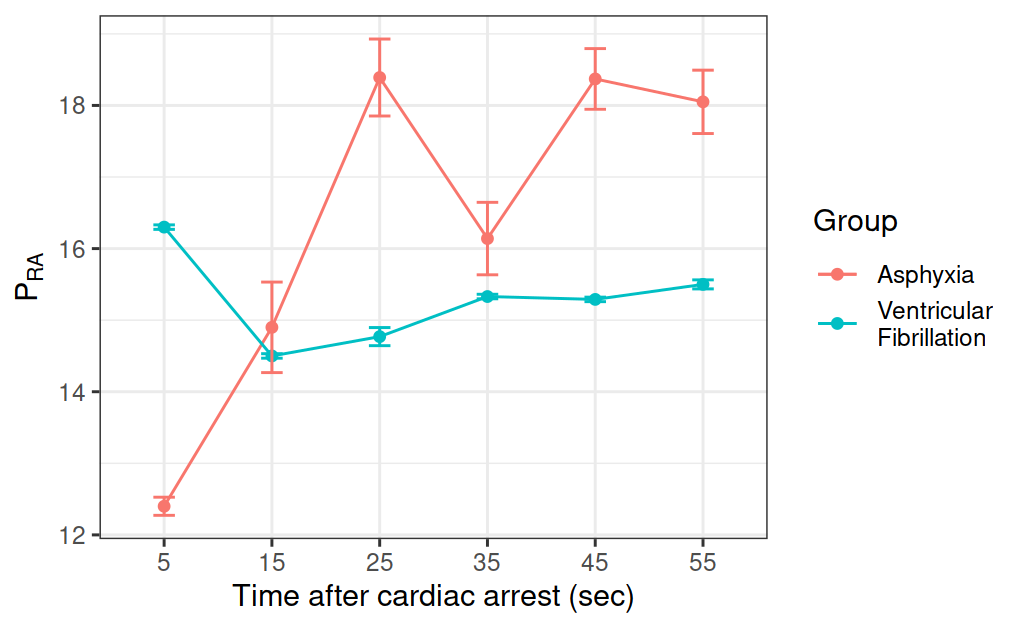

Supplement: Supplementary file 1 — Additional file 1: Figure S1. Changes in post-cardiac arrest right atrial pressure with time in the asphyxia and ventricular fibrillation group. PRA, right atrial pressure. [file 40635_2022_440_MOESM1_ESM.tiff]

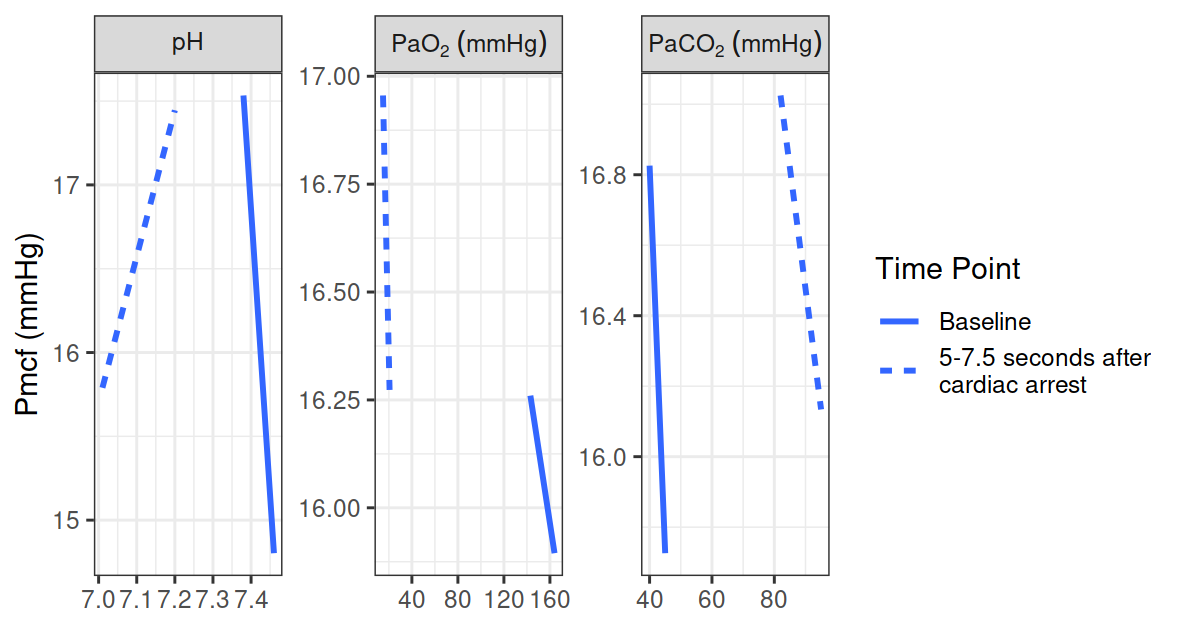

Supplement: Supplementary file 2 — Additional file 2: Figure S2. Comparison of baseline (Pmca) and post-cardiac arrest mean circulatory filling pressure in the asphyxia group demonstrating the significant effects of progressing hypoxic hypercapnia. Pmcf, mean circulatory filling pressure; PaO2, arterial partial pressure of oxygen; PaCO2, arterial partial pressure of carbon dioxide. [file 40635_2022_440_MOESM2_ESM.tiff]
